# Clinical Significance of Paraspinal Muscle Parameters as a prognostic factor for survival in Gastric Cancer Patients who underwent Curative Surgical Resection

**DOI:** 10.7150/jca.46637

**Published:** 2020-07-31

**Authors:** Wankyu Eo, Jungmi Kwon, Soomin An, Sookyung Lee, Sehyun Kim, Dongwoo Nam, Ga Young Han, Sung Il Choi, Ho-Yeon Chung

**Affiliations:** 1 Department of Medical Oncology & Hematology, College of Medicine, Kyung Hee University, Seoul, Republic of Korea.; 2 College of Nursing Science, Kyung Hee University, Seoul, Republic of Korea.; 3 Department of Clinical Oncology, College of Korean Medicine, Kyung Hee University, Seoul, Republic of Korea.; 4 Graduate School, Dankook University, Yongin, Republic of Korea; 5 Department of Acupuncture & Moxibustion Medicine, College of Korean Medicine, Kyung Hee University, Seoul, Republic of Korea.; 6 Department of Music, Chang Shin University, Changwon, Republic of Korea.; 7 Department of Surgery, College of Medicine, Kyung Hee University, Seoul, Republic of Korea.; 8 Department of Endocrinology and Metabolism, College of Medicine, Kyung Hee University, Seoul, Republic of Korea.

**Keywords:** Body Composition, Tomography, Spiral Computed, Stomach Neoplasms, Prognosis

## Abstract

**Background:** The quantitative and qualitative skeletal muscle parameters have been proposed to predict the outcome of patients with gastric cancer. However, the evidence for their association with long-term survival is still conflicting. This study aimed to investigate the effect of paraspinal muscle parameters on overall survival (OS) and disease-free survival (DFS) in patients with gastric cancer who underwent curative resection.

**Methods:** Patients with stages I or II gastric cancer who underwent curative resection between October 2006 and June 2016 were identified from electrical medical records. Paraspinal muscle area and attenuation were measured at the level of the third lumbar vertebra using computerized tomography images. For the analysis of OS and DFS, proportional hazards model was used, incorporating demographic, pathologic, laboratory, and radiologic variables.

**Results:** This study enrolled 296 patients (192 men and 104 women). In the multivariate proportional hazards model, total gastrectomy (hazard ratio [HR], 2.65; 95% Confidence interval [CI], 1.36-5.19; *p* = 0.0044), neutrophil-lymphocyte ratio (NLR) (HR, 1.27; 95% CI, 1.06-1.51; *p* = 0.0081), serum albumin level (HR, 0.16; 95% CI, 0.07-0.39; *p* < 0.0001), paraspinal muscle area adjusted for body surface area (PMA_BSA_) (HR, 3.06; 95% CI, 1.65-5.67; *p =* 0.0004), and mean attenuation in paraspinal muscle (PMMA) (HR, 3.38; 95% CI, 1.75-6.53; *p* = 0.0003) were prognostic factors for OS. Similarly, total gastrectomy (HR, 2.11; 95% CI, 1.10-4.06; *p* = 0.0243), NLR (HR, 1.25; 95% CI, 1.06-1.48; *p* = 0.0071), serum albumin level (HR, 0.22; 95% CI, 0.10-0.51; *p* = 0.0035), PMA_BSA_ (HR, 2.42; 95% CI, 1.34-4.37; *p =* 0.0035), and PMMA (HR, 3.19; 95% CI, 1.71-5.93; *p* = 0.0003) were prognostic factors for DFS.

**Conclusions:** The pretreatment paraspinal muscle parameters such as PMA_BSA_ and PMMA along with total gastrectomy, NLR, and serum albumin level could predict OS and DFS in patients with stages I or II gastric cancer who underwent curative surgical resection. Because PMA_BSA_ and PMMA are newly characterized parameters in gastric cancer, the relationship with the survival of these parameters requires further validation in further studies before they are subjected to clinical applications.

## Introduction

Stomach cancer is the third leading cause of cancer-related deaths globally. Half of all cases occur in East Asia 1, and according to the Korea Central Cancer Registry (2016) data, gastric cancer is the most frequently diagnosed malignancy in Korea 2. Although curative resection is the primary treatment for gastric cancer, the recurrence of the disease poses a problem even in early gastric cancer patients who have previously undergone curative resection. Therefore, a novel method that accurately predicts prognosis in patients with gastric cancer is needed. The tumor-related factors, including tumor size, stage, and surgical margin status 3, 4, and host-related factors, including neutrophil-lymphocyte ratio (NLR) 5-8, lymphocyte-monocyte ratio (LMR) 9, platelet-lymphocyte ratio (PLR) 10, and absolute monocyte and lymphocyte count prognostic score 11 are considered important for determining cancer recurrence and survival.

During diagnosis of gastric cancer, more than half of the patients exhibit some degree of malnutrition. The inability to maintain one's nutritional status is an important factor in determining morbidity and survival after surgery 12. Therefore, identifying malnutrition and adequate pre- and postoperative interventions for maintaining nutrition may reduce this risk. Various approaches have been used to assess the nutritional status of gastric cancer patients, such as anthropometric measurements, blood markers, and measurement of energy expenditure, validated nutritional risk scores, diet history, and body composition evaluation. The body composition analysis is considered to be a reliable approach for evaluation of muscle quantity and quality in gastric cancer patients. Muscle quantity can be estimated by a variety of techniques including dual-energy X-ray absorptiometry (DEXA), bioelectrical impedance assay (BIA), computed tomography (CT), and magnetic resonance imaging. DEXA is regarded as the most representative non-invasive method for fat-free mass measurement, but the cost of equipment, need for skilled operators, lack of portability, and exposure to ionizing radiation make clinical use difficult. The BIA is a practical, non-invasive, and an easy method to perform; however, studies that evaluated BIA gave inconsistent results. Computed tomography scan is currently considered the most accurate and non-invasive method to assess muscle mass. This approach is a regular part of the standard cancer staging; therefore, it helps avoiding additional exposure to radiation doses for measuring body composition 1. However, body composition analysis of CT images requires expensive professional software and specialized staff training for accurate measurement and analysis. Moreover, there were no established guidelines regarding standard muscle parameters to be used for measurement, appropriate image planes, and proper spinal levels.

The skeletal muscle area (SMA) is performed encompassing the psoas, multifidus, erector spinae, quadratus lumborum, and abdominal wall muscles (transversus abdominus, external and internal obliques, and rectus abdominus). CT-based measurement of SMA adjusted for height squared (such as the skeletal muscle index, SMI) at the level of the third lumbar spine (L3) is reportedly considered as a main determinant of muscle quantity. According to a recent review of the impact of CT-based measurement of SMI on the clinical outcomes, low SMI was a risk factor for both long-term and short-term survival outcomes in patients with gastrointestinal tumors 13. However, measuring SMA along the torso is difficult and time consuming. Therefore, further studies for the establishment of more convenient and accurate measurement techniques to evaluate muscle quantity are required. Muscle radiation attenuation (also known as muscle radiodensity) is a radiologic index of the muscle fat content. The advent of CT also enables explorations of changes in muscle fat avoiding invasive muscle biopsy 14. Low muscle attenuation (MA), at the level of L3, has been reported as a poor prognostic factor for survival 15-18.

Recently, a paraspinal muscle area (PMA) adjusted for height squared (such as the paraspinal muscle index, PMI) and MA in paraspinal muscle (PMMA) at the level of L3 have been reported as independent prognostic factors for survival in patients with gastrointestinal tumors 17, 19, 20. However, there is no available study considering the clinical significance of paraspinal muscle parameters on long-term survival outcome in localized gastric cancer patients.

Therefore, the aim of present study was to evaluate the clinical significance of paraspinal muscle parameters including PMI and PMMA at the level of L3 in stages I or II gastric cancer patients with a microscopically margin-negative resection (R0 resection).

## Methods

### Patient selection and study design

Patients who underwent potentially curative resection for gastric cancer between October 2006 and June 2016 in a single institution were retrospectively evaluated. This study was conducted in accordance with the Korean regulations and the Helsinki Declaration. The Institutional Review Board of Kyung Hee University Hospital at Gangdong approved the retrospective review of the electronic medical records. Written informed consent was waived for this study because of its retrospective nature. The inclusion criteria for patients were as follows: (i) diagnosed with primary gastric cancer by expert pathologists, according to Lauren's histological classification of gastric tumors 21; (ii) stages I or II, according to the 7th edition of American Joint Committee Tumor-Node-Metastasis (TNM) classification for gastric cancer 22; (iii) underwent extended lymph node dissection (D2 lymphadenectomy) and R0 resection; and (iv) underwent gastric resection by an experienced gastrointestinal surgeon (C.S.I) who participates in more than 50 gastric cancer resections a year.

The exclusion criteria for patients were as follows: (i) concurrent second malignancies or prior malignancies within the previous five years; (ii) human immunodeficiency virus-positive, evidence of acute infection, or concomitant autoimmune disease requiring immunosuppressive therapy at the time of surgery; (iii) stages 4 or 5 chronic kidney disease; (iv) without Korean Resident Registration Number; (v) without R0 resection; (vi) received chemotherapy, radiation, or any other treatment for gastric cancer before surgery; (vii) without preoperative abdominal computed tomography scans available for review; and (viii) underwent lumbar spinal intervention or surgery 23.

A total of 329 patients were initially enrolled, and 33 patients were excluded for the following reasons: (i) eighteen patients because of the loss of preoperative CT records or poor image quality; (ii) seven Russian patients considering ethnic difference that may have affected muscle quantity or quality parameters; (iii) three patients because gastric cancer and other malignant tumors were simultaneously diagnosed; (iv) two patients without R0 resection; (v) one patient with stage 5 chronic kidney disease; (vi) one patient with acute infection; and (vii) one patient because he had a history of surgery for lumbar disease.

### Clinical variables

Records of demographic and clinical variables such as age, sex, site of tumor, size of tumor, type of gastrectomy, TNM stage, Lauren classification, lymphatic/vascular/perineural invasion, adjuvant chemotherapy, anemia, serum albumin level, NLR, LMR, and PLR were collected and analyzed. Because there were no age restrictions, elderly patients were also included in this study. Analysis of blood test results was done on tests performed within seven days before surgery. If there was more than one preoperative test result, the test result closest to the date of surgery was selected. The diagnoses of anemia in men and women were based on hemoglobin levels lower than 13 g/dL and 12 g/dL, respectively.

### Body composition

**Figure 1:**
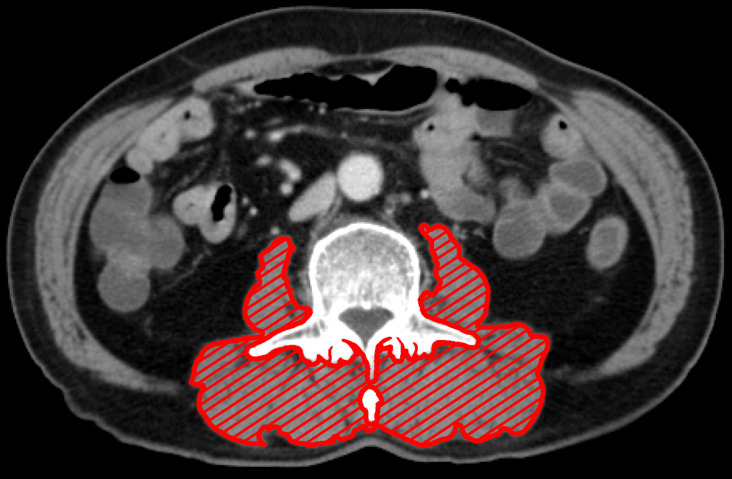
The cross-sectional area at L3 of paraspinal muscle (including erector spinae, multifidus, psoas, and quadratus lumborum). Paraspinal muscle is highlighted in red.

Computed tomography images taken within 30 days before surgical resection were analyzed. After identification of the L3 landmark, corresponding single axial image was extracted and saved as a DICOM image file 24. SliceOmatic software (ver. 5.0) was used to measure the patient's body composition. Hounsfield unit (HU) threshold (-29 to +150) was used to identify and quantify PMA and PMMA. For measurement of the PMA, the erector spinae, multifidus, psoas, and quadratus lumborum were encompassed (Fig. 1). The PMI was calculated by dividing PMA by the square of the patient's height in meters. Finally, PMMA at the level of L3 was calculated; Region of interest was characterized as all pixels within muscle HU range (-29 to +150 HU). All the measurements were performed by an experienced nurse, and the tagged image file was reconfirmed by an experienced physician before entering it in the database. All measurements were performed under the supervision of a musculoskeletal radiologist. Before statistical analysis, muscle area and attenuation were categorized with sex-specific cutoff points.

### Statistical analysis

Overall survival (OS) was defined as the time from the date of surgery to the date of death or last follow-up. Disease-free survival (DFS) was defined as the time from the date of surgery to the date of relapse, death, or last follow-up. Patients who did not experience relapse or death were censored at the last follow-up. Curves for OS and DFS were depicted using the Kaplan-Meier method, and differences between survival curves were tested for statistical significance using the log-rank test. The continuous variables without well-known cutoff point such as size of tumor, NLR, LMR, PLR, height, and body weight were dichotomized using R packages (maxstat) before analysis.

The Cox proportional hazard model was used to identify the most valuable prognostic factors for OS and DFS. The continuous variables without well-known cutoff points were not dichotomized before analysis. Only those variables that were compatible with proportional hazard assumption using the Schoenfeld residual test were analyzed using the univariate Cox model. Those variables with *p* < 0.05 in the univariate Cox model were further analyzed using the multivariate Cox model. The Harrell's concordance statistics for Cox model was performed to measure discriminative capacity. The variance inflation factor (VIF) was calculated for diagnosis of multicollinearity.

All *p*-values presented were 2-sided, and statistical significance was declared at *p* < 0.05. Data were analyzed using R packages and MedCalc (Ver. 19.2, MedCalc Software Ltd, Belgium).

## Results

### Baseline clinical characteristics of patients

Baseline patient characteristics are shown in Table 1. The median age of the patients was 60 years. There were 224 (75.7%) patients in stage I and 72 (24.3%) in stage II. Total gastrectomy was performed in 51 (17.2%) patients. The intestinal type by Lauren's classification was the most common type (53.4%). Anemia was found in 89 (30.1%) of the patients enrolled. The median of serum albumin level was 4.2 g/dL. The medians of NLR, LMR, and PLR were 1.9, 4.4, and 117.2, respectively.

### Paraspinal muscle parameters

**Figure 2:**
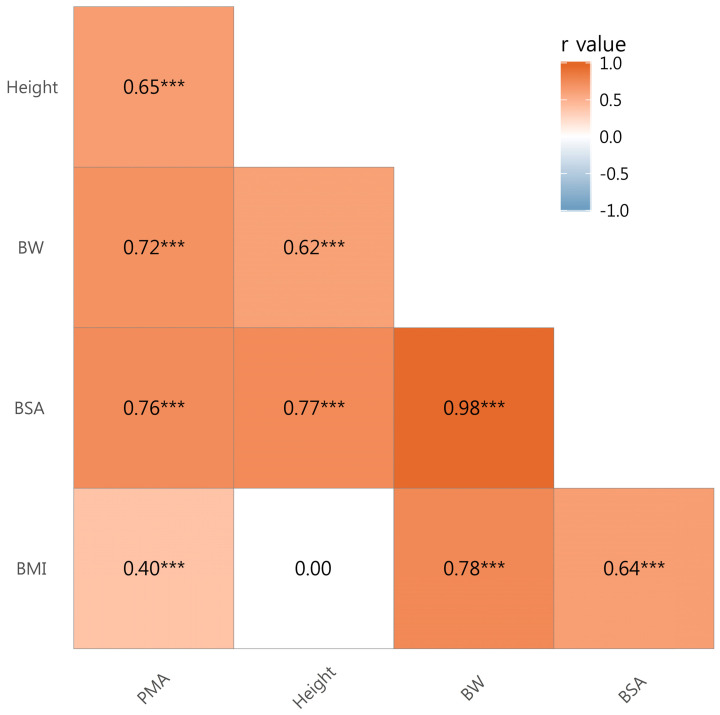
Correlation Coefficients by Pearson's product-moment correlation. Abbreviations: BW, body weight; BSA, body surface area; BMI, body mass index; PMA, paraspinal muscle area; *** *p* value < 0.001.

There were significant correlations between PMA and height (*r* = 0.65, *p* < 0.001), body weight (*r* = 0.72, *p* < 0.001), body surface area (BSA) (*r* = 0.76, *p* < 0.001), and body mass index (BMI) (*r* = 0.40, *p* < 0.001) (Fig. 2). Therefore, PMA was further adjusted for body weight, BSA, and BMI forming PMA_BW_, PMA_BSA_, and PMA_BMI_, respectively.

As there was a significant difference in the medians of PMI, PMA_BW_, PMA_BSA_, PMA_BMI_, and PMMA between sexes (*p* < 0.0001 in all variable), the threshold values of these parameters were determined with sex-specific cutoff points (Table 2).

### Paraspinal muscle parameters and survival

With a median follow-up of 80.5 months (range, 0.9-145.5 months), the Kaplan-Meier method followed by the log-rank test revealed that there was a significant difference in OS in variables such as age (*p* < 0.0001), size of tumor (*p* = 0.0103), stage (*p* = 0.0152), total gastrectomy (*p* = 0.0014), lymphatic invasion (*p* = 0.0133), NLR (*p* < 0.0001), LMR (*p* < 0.0001), PLR (*p* = 0.0277), anemia (*p* = 0.0017), hypoalbuminemia (*p* < 0.0001), height (*p* = 0.0028), body weight (*p* = 0.0013), BSA (*p* = 0.0063), PMI (*p* = 0.0008), PMA_BW_ (*p* = 0.0383), PMA_BSA_ (*p* < 0.0001), PMA_BMI_ (*p* = 0.0014), and PMMA (*p* < 0.0001) (Table 3).

Only those variables that were compatible with proportional hazard assumption using the Schoenfeld residual test were analyzed using the univariate Cox model. Therefore, lymphatic invasion and perineural invasion were excluded from analysis of prognostic factor for OS; in addition, lymphatic invasion, perineural invasion and BSA were excluded from analysis of prognostic factor for DFS. In the univariate Cox proportional hazards model for OS, variables such as age, stage, total gastrectomy, NLR, LMR, anemia, serum albumin level, PMI, PMA_BW_, PMA_BSA_, PMA_BMI_, and PMMA were significant. However, using the multivariate Cox model, only variables such as total gastrectomy (hazard ratio [HR], 2.65; 95% Confidence interval [CI], 1.36-5.19; *p* = 0.0044), NLR (HR, 1.27; 95% CI, 1.06-1.51; *p* = 0.0081), serum albumin level (HR, 0.16; 95% CI, 0.07-0.39; *p* < 0.0001), PMA_BSA_ (HR, 3.06; 95% CI, 1.65-5.67; *p =* 0.0004), and PMMA (HR, 3.38; 95% CI, 1.75-6.53; *p* = 0.0003) were significant. The Harrell's concordance statistics for Cox model was 0.8085, indicating excellent discrimination. The VIFs for total gastrectomy, NLR, serum albumin level, PMA_BSA_, and PMMA were 1.15, 1.04, 1.14, 1.04, and 1.01, respectively; therefore, there was no significant collinearity between the variables (Table 4).

Using the univariate Cox model for DFS, the same variables which proved significant in the OS analysis, except PMA_BW_, were identified as significant. However, using the multivariate Cox model, total gastrectomy (HR, 2.11; 95% CI, 1.10-4.06; *p* = 0.0243), NLR (HR, 1.25; 95% CI, 1.06-1.48; *p* = 0.0071), serum albumin level (HR, 0.22; 95% CI, 0.10-0.51; *p* = 0.0035), PMA_BSA_ (HR, 2.42; 95% CI, 1.34-4.37; *p =* 0.0035), and PMMA (HR, 3.19; 95% CI, 1.71-5.93; *p* = 0.0003) were significant. The Harrell's concordance statistics for Cox model was 0.7716, indicating acceptable discrimination. The VIFs for total gastrectomy, NLR, serum albumin level, PMA_BSA_, and PMMA were 1.14, 1.02, 1.14, 1.03, and 1.01, respectively; therefore, there was no significant collinearity between the variables (Table 5).

## Discussions

The aim of present study was to evaluate the clinical significance of paraspinal muscle parameters at the level of L3 in stages I or II gastric cancer patients, and we found that paraspinal muscle parameters (such as PMA_BSA_ and PMMA) could predict survival along with total gastrectomy, NLR, and serum albumin level.

In our study, paraspinal muscle was composed of multifidus, erector spinae, psoas, and quadratus lumborum as previously reported 15, 25, 26, considering the time required for the analysis and reproducibility. However, the definition of paraspinal muscle has been somewhat heterogeneous among various studies. In studies by Dohzono and Deng, it was suggested that paraspinal muscle was composed of multifidus and erector spinae 19, 20. In other studies, multifidus and erector spinae combined with either psoas muscle 27, quadratus lumborum 17, or psoas muscle/quadratus lumborum 15, 25, 26 were defined as paraspinal muscles.

In our study, we found that PMA at the level of L3 is correlated with height, body weight, BSA, and BMI. These findings are in line with those of a previous study by Yoshizumi that showed that SMA at the level of L3 was significantly associated with the same variables as those used in our study 28. Therefore, in our study, PMA was adjusted for height squared, body weight, BSA, and BMI forming PMI, PMA_BW_, PMA_BSA_, and PMA_BMI_, respectively.

The role of PMI as a prognostic factor for survival has been reported recently in gastrointestinal tumors. In Hacker's study on advanced gastric and gastroesophageal junction cancers, PMI was shown to be a significant predictor for OS (*p* = 0.003) but not for DFS using the multivariate Cox model 17. In our study, PMI was a significant prognostic factor for OS and DFS when applying univariate Cox model; however, PMI was not an independent prognostic factor for OS or DFS when the multivariate Cox model was used. It is believed that the difference in the cutoff points, the definition of paraspinal muscles, the stage of the tumor, and the location of tumor may have resulted in the inconsistent findings among studies.

Body surface area has been used to estimate metabolic rate dates since the late 19th century. Currently, cardiac output, glomerular filtration rate and pulmonary function tests, chemotherapy doses, fluid resuscitation, and calories needed are frequently expressed based on BSA 29. In our study, there was a significant difference in OS and DFS according to BSA when applying the log-rank test. In addition, BSA was correlated with PMA, as has been reported previously 28, 30. Therefore, we evaluated the clinical significance of PMA adjusted for BSA (PMA_BSA_). Because PMA_BSA_ was dependent on the sex in our study, PMA_BSA_ was dichotomized with sex-specific cutoff points. In our study, PMA_BSA_ was a significant prognostic factor for both OS and DFS using the multivariate Cox model. Similarly, Chang also found that appendicular lean mass adjusted for BSA was more accurate in predicting low muscle function than are height squared- and weight-adjusted indices 30. Because there is no available study on the clinical significance of PMA_BSA_ in gastrointestinal tumors, further studies on the value of PMA_BSA_ is needed for validation of our findings.

Skeletal muscle contains lipid droplets within the myocytes as well as intermuscular adipocytes. The MA is a radiologic index of muscle fat content, and it is inversely related to muscle fat content 14. When reporting MA, it is recommended to use predefined HU ranges to demarcate intermuscular adipose tissue (usually -190 to -30 HU) and muscle tissue (usually -29 HU to +150 HU). In previous studies, MA has been measured in skeletal muscle areas encompassing multiple muscles along the torso (such as psoas, multifidus, erector spinae, quadratus lumborum, and abdominal wall muscles). The mean skeletal muscle attenuation (SMMA) below threshold was a poor prognostic factor for OS in patients with gastrointestinal tumors 15, 17, 18. Therefore, SMMA is suggested to be an important biomarker for survival in gastrointestinal tumors, which still needs further studies for validation.

In our study, instead of measuring MA in skeletal muscle along the torso, we measured MA in paraspinal muscles (PMMA). In this study, there was a significant difference in median values of PMMA according to sex (*p* < 0.0001), and a significant correlation between PMMA and BMI (*r* = -0.16, *p* = 0.0046). The significant correlation between MA and sex or BMI has been suggested previously 15, 17, 20, 31. Therefore, when determining the PMMA threshold for survival, we initially planned to use both sex-and BMI-specific cutoff points as has been reported by Martin 15. However, in a subgroup analysis, we found that there was no significant correlation between PMMA and BMI in female patients. Then, the PMMA threshold for survival was determined with sex-specific cutoff points as has been reported by Dohzono 20. In our study, we found that PMMA was a significant prognostic factor for both OS and DFS using the multivariate Cox models. Our result is compatible with that of Dohzono who showed that lower PMMA was an independent poor prognostic factor in patients with gastrointestinal cancer with spine metastasis 20. Therefore, measuring PMMA could be a promising alternative to measuring SMMA along the torso. However, as there are only few available studies on the clinical value of PMMA in malignancies, validation of our results by further evaluation is required.

Pathological variation in MA reflects excess fat deposition in the tissue, and is observed in people with elderly, obesity, and cancer. A poor prognosis is predicted by the presence of reduced mean MA values in patients with these conditions 14. When dichotomizing our cohort into patients with PMMA-low and PMMA-high groups, there was a significant difference between two groups in terms of age (*p* <0.0001), sex (*p* < 0.0001), total gastrectomy (*p* = 0.0250), LMR (*p* = 0.0008), serum albumin level (*p* = 0.0011), PMI (*p* = 0.0477), and PMA_BMI_ (*p* = 0.0222) (Table 6); therefore, relative older age, male predominance, higher total gastrectomy, lower LMR, lower PMI, and lower PMA_BMI_ may have affected the poor prognosis in PMMA-low group. Although the exact mechanism underlying the poor survival in patients with lower PMMA needs further study, in our cohort, diverse independent prognostic factors, including demographic, inflammatory, or nutritional status, may have affected the dismal prognosis in this group.

In our study, total gastrectomy was a significant factor for OS and DFS by multivariate Cox analysis. In a subgroup analysis, when dichotomizing our cohort into patients with total gastrectomy and partial gastrectomy, there was a significant difference between two groups in terms of age (*p* = 0.002), site of tumor (*p* = 0.001), and size of tumor (*p* = 0.001); therefore, relative older age, proximal location, and increased tumor size may have affected the poor prognosis in patients who underwent total gastrectomy. In contrast, there was no significant difference in serum albumin level or paraspinal muscle parameters (such as PMA, PMI, PMA_BSA_, and PMMA) between two groups; therefore, baseline nutritional parameters did not influence the clinical course of patients.

In our study, NLR was a significant prognostic factor for OS and DFS using the multivariate Cox model. Our results are compatible with Mellor's meta-analysis results; Mellor also showed that NLR was an important prognostic determinant for both OS and DFS after R0 resection of gastric cancer 32.

Serum albumin level has been considered as a significant nutritional marker for survival in gastric cancer 8, 33, 34. In this study, we also found that serum albumin level was a prognostic factor for OS and DFS using the multivariate Cox model.

The strength of our study is that, to the best of our knowledge, this is the first study to evaluate the prognostic significance of PMMA in patients with early stage gastric cancer who underwent curative gastric resection. In addition, we evaluated the clinical significance of diverse paraspinal muscle parameters (such as PMA adjusted for body weight, BSA, and BMI) in patients with early stage gastric cancer, and this also could be the first trial. In this study, we found that two pretreatment paraspinal muscle parameters (such as PMMA and PMA_BSA_) could independently predict long-term outcomes (such as OS and DFS) along with total gastrectomy, NLR, and serum albumin level. Finally, another strength of our study is that, for consistency, we included only those patients who underwent gastric resection by an experienced gastrointestinal surgeon who participates in more than 50 gastric tumor resections a year.

This study has some limitations; hence, the results of the study should be interpreted carefully. First, this study was performed retrospectively; therefore, missing data including CT images was inevitable, and it may have affected the results. Second, as this was a retrospective study, we did not have an opportunity to provide special interventions to patients with lower than threshold level of PMA_BSA_ and PMMA to improve postoperative outcomes. Third, although both random errors and potential biases were controlled from planning through to implementation of the study, the lack of validation with an independent cohort is another limitation of our study. Regarding public database, we have a limitation issue to use CT images for review in Korean population. In addition, worldwide databases have an ethnicity issue. Based on the result of this study, we can perform prospective study with independent external validation group in the next step of this study.

In conclusion, we found that PMA_BSA_, PMMA, total gastrectomy, NLR, and serum albumin level were significant determinants for both OS and DFS. The concordance statistics for the same covariates were excellent during discrimination for OS and acceptable during discrimination for DFS. Using VIFs, there was no significant collinearity between covariates in the Cox model for survival analysis. Because PMA_BSA_ and PMMA are newly characterized parameters in gastric cancer, the relationship with the survival of these parameters requires further validation in further studies before they are subjected to clinical applications.

## Figures and Tables

**Table 1:** Characteristic of patients with gastric cancer (*n* = 296)

Variables	Median (IQR), or *n* (%)
**Age (years)**	60.0 (52.0-68.0)
**Sex**	
Male	192 (64.1)
Female	104 (35.9)
**Site of tumor**	
Upper	28 (9.5)
Middle	129 (43.6)
Lower	136 (45.9)
Diffuse	3 (1.0)
**Size of tumor (cm)**	2.6 (1.8-4.0)
**TNM stage**	
I	224 (75.7)
II	72 (24.3)
**Total gastrectomy**	
Yes	51 (17.2)
No	245 (82.8)
**Lauren classification**	
Intestinal	157 (53.4)
Diffuse	69 (23.5)
Mixed	55 (18.7)
Unknown	13 (4.4)
**Lymphatic invasion**	
No	239 (80.7)
Yes	57 (19.3)
**Vascular invasion**	
No	290 (98.0)
Yes	6 (2.0)
**Perineural invasion**	
No	283 (95.6)
Yes	13 (4.4)
**Adjuvant chemotherapy**	
No	225 (76.0)
Yes	71 (24.0)
Anemia*	
No	207 (69.9)
Yes	89 (30.1)
**Serum albumin (g/dL)**	4.2 (4.0-4.3)
**NLR**	1.9 (1.4-2.5)
**LMR**	4.4 (3.4-5.5)
**PLR**	117.2 (93.6-147.4)

Abbreviations: IQR, interquartile range; TNM, tumor-node-metastasis; NLR, neutrophil-lymphocyte ratio; LMR, lymphocyte-monocyte ratio; PLR, platelet-lymphocyte ratio. * The cutoff point is 12 g/dL in female patients and 13 g/dL in male patients.

**Table 2:** Threshold values of paraspinal muscle area adjusted for height squared, body weight, body surface area, body mass index, and mean attenuation within paraspinal muscle according to the sex

	Threshold values*	
	Male (n = 192)	Female (n = 104)
PMI	29.76	23.70
PMA_BW_	0.97	0.94
PMA_BSA_	38.50	32.41
PMA_BMI_	2.68	2.05
PMMA	48.07	31.16

Abbreviations: PMI, paraspinal muscle index (also known as paraspinal muscle area adjusted for height squared); PMA, paraspinal muscle area; PMA_BW_, PMA adjusted for body weight; PMA_BSA_, PMA adjusted for body surface area; PMA_BMI_, PMA adjusted for body mass index; PMMA, mean attenuation within paraspinal muscle. * The threshold is determined using R packages (maxstat).

**Table 3:** The overall survival and disease-free survival values according to the clinicopathologic variables

	*n*	5-year OS (%)	*p*-value	5-year DFS (%)	*p*-value
**Age (years)**					
<65	181	94.8	<0.0001	92.6	<0.0001
≥65	115	79.0		78.2	
**Sex**					
Male	192	86.8	0.3720	84.8	0.1840
Female	104	90.9		91.0	
Size of tumor (cm)*					
≤4.5	243	91.0	0.0103	89.3	0.0304
>4.5	53	78.1		76.2	
**TNM stage**					
I	224	92.1	0.0152	90.7	0.0176
II	72	78.3		75.4	
**Total gastrectomy**					
No	245	90.7	0.0014	88.6	0.0060
Yes	51	78.2		78.4	
**Lauren classification**					
Intestinal	157	90.8	0.7730	86.2	0.9110
Others	139	86.8		87.8	
**Lymphatic invasion**					
No	239	91.6	0.0133	89.9	0.0383
Yes	57	76.8		75.0	
**Vascular invasion**					
No	290	89.1	0.1430	87.4	0.1860
Yes	6	66.7		66.7	
**Perineural invasion**					
No	283	89.6	0.0917	87.8	0.1340
Yes	13	69.2		69.2	
NLR*					
≤3.26	262	92.4	<0.0001	90.9	<0.0001
>3.26	33	60.3		57.0	
LMR*					
≤2.79	35	53.0	<0.0001	49.9	<0.0001
>2.79	260	93.6		92.0	
PLR*					
≤188.82	261	90.4	0.0277	88.4	0.0652
>188.82	34	78.1		78.2	
**Anemia§**					
No	207	91.8	0.0017	90.4	0.0032
Yes	89	81.3		79.0	
**Hypoalbuminemia**					
No	282	91.4	<0.0001	89.6	<0.0001
Yes	14	35.7		35.7	
Height (cm)*					
≤151.0	36	78.8	0.0028	76.8	0.0103
>151.0	260	90.4		88.4	
Body weight (kg)*					
≤53.8	63	81.8	0.0013	81.8	0.0068
>53.8	233	90.6		88.4	
BSA (m^2^)*					
≤1.48	37	80.9	0.0063	80.9	0.0190
>1.48	259	89.9		87.9	
**BMI (kg/m^2^)**					
<18.5	16	87.1	0.2190	87.1	0.3050
≥18.5	280	88.8		87.0	
**BMI (kg/m^2^)**					
<25	190	86.7	0.1150	85.1	0.0994
≥25	106	92.2		90.3	
**PMI (cm^2^/m^2^)¶**					
Low	206	84.9	0.0008	83.5	0.0010
High	90	97.8		95.2	
**PMA_BW_¶**					
Low	65	82.5	0.0383	82.5	0.1040
High	231	90.4		88.2	
**PMA_BSA_¶**					
Low	66	78.4	<0.0001	78.4	0.0007
High	230	91.7		89.5	
**PMA_BMI_¶**					
Low	50	79.8	0.0014	79.8	0.0064
High	246	90.6		88.5	
**PMMA (HU)¶**					
Low	104	79.3	<0.0001	76.4	<0.0001
High	192	93.9		92.8	

Abbreviations: OS, overall survival; DFS, disease-free survival; TNM, tumor-node-metastasis; NLR, neutrophil-lymphocyte ratio; LMR, lymphocyte-monocyte ratio; PLR, platelet-lymphocyte ratio; BSA, body surface area; BMI, body mass index; PMI, paraspinal muscle index (also known as paraspinal muscle area adjusted for height squared); PMA, paraspinal muscle area; PMA_BW_, PMA adjusted for body weight; PMA_BSA_, PMA adjusted for BSA; PMA_BMI_, PMA adjusted for BMI; PMMA, mean attenuation within paraspinal muscle; HU, Hounsfield unit. * The cutoff point is determined by using R packages (maxstat). § The cutoff point is 12 g/dL in female patients and 13 g/dL in male patients. ¶ The threshold is determined with sex-specific cutoff point (Table 2). Curves for OS and DFS were depicted using the Kaplan-Meier method, and differences between survival curves were tested for statistical significance using the log-rank test.

**Table 4:** Univariate and multivariate Cox proportional hazards regression analysis of overall survival

Variable	Overall survival			
	Univariate		Multivariate	
	HR (95% CI)	*p*-value	HR (95% CI)	*p*-value
Age (years) (<65 vs. ≥65)	4.19 (2.18-8.04)	<0.0001		
Sex (Male vs. Female)	0.74 (0.39-1.43)	0.3741		
Size of tumor (cm)	1.09 (0.99-1.19)	0.0787		
TNM stage (I vs. II)	2.10 (1.14-3.87)	0.0177		
Total gastrectomy (No vs. Yes)	2.71 (1.43-5.13)	0.0022	2.65 (1.36-5.19)	0.0044
Lauren (Others vs. Intestinal)	1.09 (0.60-1.99)	0.7734		
Vascular invasion (No vs. Yes)	2.77 (0.67-11.45)	0.1603		
NLR	1.36 (1.18-1.56)	<0.0001	1.27 (1.06-1.51)	0.0081
LMR	0.71 (0.56-0.90)	0.0048		
PLR	1.00 (1.00-1.01)	0.2337		
Anemia (No vs. Yes)*	2.52 (1.39-4.59)	0.0024		
Serum albumin	0.11 (0.05-0.23)	<0.0001	0.16 (0.07-0.39)	<0.0001
BSA	0.18 (0.03-1.05)	0.0564		
BMI	0.92 (0.83-1.01)	0.0896		
PMI (High vs. Low)	5.83 (1.80-18.84)	0.0032		
PMA_BW_ (High vs. Low)	1.92 (1.02-3.59)	0.0418		
PMA_BSA_ (High vs. Low)	3.12 (1.71-5.68)	0.0002	3.06 (1.65-5.67)	0.0004
PMA_BMI_ (High vs. Low)	2.67 (1.42-4.99)	0.0022		
PMMA (High vs. Low)	4.41 (2.30-8.46)	<0.0001	3.38 (1.75-6.53)	0.0003

Abbreviations: HR, hazard ratio; CI, confidence interval; TNM, tumor-node-metastasis; Lauren, Lauren classification; NLR, neutrophil-lymphocyte ratio; LMR, lymphocyte-monocyte ratio; PLR, platelet-lymphocyte ratio; BSA, body surface area; BMI, body mass index; PMI, paraspinal muscle index (also known as paraspinal muscle area adjusted for height squared); PMA, paraspinal muscle area; PMA_BW_, PMA adjusted for body weight; PMA_BSA_, PMA adjusted for BSA; PMA_BMI_, PMA adjusted for BMI; PMMA, mean attenuation within paraspinal muscle. * The cutoff point is 12 g/dL in female patients and 13 g/dL in male patients. The Harrell's concordance statistics for Cox model is 0.8085, indicating excellent discrimination. The variance inflation factors for total gastrectomy, NLR, serum albumin level, PMA_BSA_, and PMMA are 1.15, 1.04, 1.14, 1.04, and 1.01, respectively.

**Table 5:** Univariate and multivariate Cox proportional hazards regression analysis of disease-free survival

Variable	Disease-free survival				
	Univariate		Multivariate		
	HR (95% CI)	*p*-value	HR (95% CI)		*p*-value
Age (years) (<65 vs. ≥65)	3.45 (1.89-6.31)	<0.0001			
Sex (Male vs. Female)	0.65 (0.34-1.23)	0.1872			
Size of tumor (cm)	1.07 (0.98-1.17)	0.1468			
Total gastrectomy (No vs. Yes)	2.34 (1.25-4.38)	0.0076	2.11 (1.10-4.06)		0.0243
TNM stage (I vs. II)	2.01 (1.17-3.62)	0.0200			
Lauren (Others vs. Intestinal)	0.97 (0.55-1.72)	0.9114			
Vascular invasion (No vs. Yes)	2.52 (0.61-10.38)	0.2019			
NLR	1.33 (1.17-1.52)	<0.0001	1.25 (1.06-1.48)		0.0071
LMR	0.69 (0.55-0.87)	0.0016			
PLR	1.00 (1.00-1.01)	0.2467			
Anemia (No vs. Yes)*	2.31 (1.30-4.09)	0.0042			
Serum albumin	0.14 (0.06-0.28)	<0.0001	0.22 (0.10-0.51)		0.0035
BMI	0.92 (0.84-1.01)	0.0858			
PMI (High vs. Low)	4.74 (1.70-13.20)	0.0029			
PMA_BW_ (High vs. Low)	1.65 (0.90-3.06)	0.1079			
PMA_BSA_ (High vs. Low)	2.61 (1.46-4.65)	0.0012	2.42 (1.34-4.37)		0.0035
PMA_BMI_ (High vs. Low)	2.29 (1.24-4.23)	0.0080			
PMMA (High vs. Low)	4.16 (2.25-7.68)	<0.0001	3.19 (1.71-5.93)		0.0003
					

Abbreviations: HR, hazard ratio; CI, confidence interval; TNM, tumor-node-metastasis; Lauren, Lauren classification; NLR, neutrophil-lymphocyte ratio; LMR, lymphocyte-monocyte ratio; PLR, platelet-lymphocyte ratio; BMI, body mass index; PMI, paraspinal muscle index (also known as paraspinal muscle area adjusted for height squared); PMA, paraspinal muscle area; PMA_BW_, PMA adjusted for body weight; PMA_BSA_, PMA adjusted for BSA; PMA_BMI_, PMA adjusted for BMI; PMMA, mean attenuation within paraspinal muscle. * The cutoff point is 12 g/dL in female patients and 13 g/dL in male patients. The Harrell's concordance statistics for Cox model is 0.7716, indicating acceptable discrimination. The variance inflation factors for total gastrectomy, NLR, serum albumin level, PMA_BSA_, and PMMA are 1.14, 1.02, 1.14, 1.03, and 1.01, respectively.

**Table 6:** Characteristics according to the mean attenuation within paraspinal muscles

Variables	PMMA-low group (*n* = 104)	PMMA-high group (*n* = 192)	*p*-value
	Median (IQR), or *n* (%)	Median (IQR), or *n* (%)	
**Age (years)**	65.0 (56.5-72.0)	58.0 (50.0-66.0)	< 0.0001
**Sex**			
Male	93 (89.4)	99 (51.6)	< 0.0001
Female	11 (10.6)	93 (48.4)	
**Size of tumor (cm)**	2.7 (2.0-4.5)	2.5 (1.8-3.7)	0.2210
**TNM stage**			
I	72 (69.2)	152 (79.2)	0.0655
II	32 (30.8)	40 (20.8)	
**Total gastrectomy**			
No	79 (76.0)	166 (86.5)	0.0250
Yes	25 (24.0)	26 (13.5)	
**Lauren classification**			
Intestinal	63 (60.6)	94 (49.0)	0.0673
Others	41 (39.4)	98 (51.0)	
**Lymphatic invasion**			
No	79 (76.0)	160 (83.3)	0.1640
Yes	25 (24.0)	32 (16.7)	
**Neural invasion**			
No	97 (93.3)	186 (96.9)	0.2330
Yes	7 (6.7)	6 (3.1)	
**Vascular invasion**			
No	102 (98.1)	188 (97.9)	1.0000
Yes	2 (1.9)	4 (2.1)	
**NLR**	2.0 (1.5-2.7)	1.8 (1.3-2.4)	0.0525
**LMR**	4.0 (3.1-5.3)	4.6 (3.7-5.6)	0.0008
**PLR**	114.6 (86.7-146.3)	117.6 (96.2-148.4)	0.2530
**Anemia§**			
No	68 (65.4)	139 (72.4)	0.2329
Yes	36 (34.6)	53 (27.6)	
**Albumin (g/dL)**	4.1 (3.9-4.3)	4.2 (4.0-4.4)	0.0011
**BMI (kg/m^2^)**			
<25	61 (58.7)	129 (67.2)	0.1630
≥25	43 (41.3)	63 (32.8)	
**PMI (cm^2^/m^2^)¶**			
Low	80 (76.9)	126 (65.6)	0.0477
High	24 (23.1)	66 (34.4)	
**PMA_BW_¶**			
Low	22 (21.2)	43 (22.4)	0.8830
High	82 (78.8)	149 (77.6)	
**PMA_BSA_¶**			
Low	30 (28.8)	36 (18.8)	0.0571
High	74 (71.2)	156 (81.2)	
**PMA_BMI_¶**			
Low	25 (24.0)	25 (13.0)	0.0222
High	79 (76.0)	167 (87.0)	

Abbreviations: PMMA, mean attenuation within paraspinal muscle; IQR, interquartile range; TNM, tumor-node-metastasis; NLR, neutrophil-lymphocyte ratio; LMR, lymphocyte-monocyte ratio; PLR, platelet-lymphocyte ratio; BMI, body mass index; PMI, paraspinal muscle index (also known as paraspinal muscle area adjusted for height squared); PMA, paraspinal muscle area; PMA_BW_, PMA adjusted for body weight; PMA_BSA_, PMA adjusted for body surface area; PMA_BMI_, PMA adjusted for BMI. § The cutoff point is 12 g/dL in female patients and 13 g/dL in male patients. ¶ The threshold is determined with sex-specific cutoff point (Table 2).
